# STEM gender stereotypes from early childhood through adolescence at informal science centers

**DOI:** 10.1016/j.appdev.2020.101109

**Published:** 2020

**Authors:** Luke McGuire, Kelly Lynn Mulvey, Eric Goff, Matthew J. Irvin, Mark Winterbottom, Grace E. Fields, Adam Hartstone-Rose, Adam Rutland

**Affiliations:** a University of Exeter, UK; b North Carolina State University, NC, USA; c University of South Carolina, SC, USA; d Cambridge University, UK; e Riverbanks Zoo & Gardens, SC, USA

**Keywords:** STEM stereotypes, Gender stereotypes, Informal settings

## Abstract

Stereotypes about science, technology, engineering and mathematics (STEM) are associated with reduced STEM engagement amongst girls and women. The present study examined these stereotypes from early childhood through adolescence within informal science learning sites (ISLS; science museums, zoos, aquariums). Further, the study explored whether interactions with male or female educators influenced STEM stereotypes. Participants (*n* = 997, female = 572) were ISLS visitors in the UK and USA who either interacted with an educator, or no educator. With age participants were more likely to report that “both boys and girls” are “usually”, “should” be, and “can” be good at STEM. Independent of age, male participants reported that their own gender group “should” be good at STEM. Educator interactions did not influence stereotype responses. These results highlight early childhood as a key developmental window in which to challenge ideas about who can and should be proficient in STEM.

## Highlights

• Gender stereotypes about science, technology, engineering and mathematics (STEM) ability emerge in early childhood
• Adolescents are more likely to say that both boys and girls should, can and usually are good at STEM
• Boys are more likely than girls to say that their own gender group ‘should’ be good at STEM
• Stereotypes do not change based on an interaction with an educator (male or female) in an informal science learning site

## Introduction

Gender stereotypes about who *can be*, *should be* and *is* usually good at science, technology, engineering and mathematics (STEM) have long-lasting consequences for engagement with and motivation towards STEM domains. These stereotypes emerge in childhood (Cvencek, Meltzoff, & Greenwald, 2011) and are reinforced in adolescence by the presence of male teachers in STEM subjects and an imbalanced classroom gender composition (Riegle-Crumb, Moore, & Buontempo, 2017). Crucially, these stereotypes persist in to the work place and broader society, making an impact on representation of women in the STEM fields. There is a need to challenge ideas about STEM ability based on gender, which contribute to disparities in gender representation in higher education and employment. For example, in the UK, women make up only 22% of the STEM workforce (WISE, 2018) and in the US, women make up only 24% of STEM workforce (Noonan, 2017).

Informal STEM learning settings (ISLS; e.g., museums, science centers, zoos, aquariums) provide an opportunity for children and adolescents to engage with STEM outside of the classroom, but also to interact with counter-stereotypical educators (i.e. women, people from ethnic minority backgrounds). The present study, for the first time, examines STEM gender stereotypes across development from early childhood to adolescence, exhibited in informal STEM learning settings. Second, this study also examines the potential influence of interactions with informal STEM learning educators on children's and adolescent's STEM gender stereotypes.

### STEM gender stereotypes

Gender is one social category where stereotypes begin to emerge from an early age. Gender is a central factor that children use to categorize and compare themselves in relation to others from the preschool years (Liben & Bigler, 2002; Renno & Shutts, 2015). At the same time that children become aware of these categories, their broader interactions with adults can reinforce gender-typed behaviors (Ruble, Martin, & Berenbaum, 2006). Consequently, from as young as two-years-old children understand gender labels (Ruble et al., 2006) and begin to develop ideas about gender groups that are generalized in the form of stereotypes shortly afterwards (Mulvey, Hitti, & Killen, 2010). By two-and-a-half years, children are aware of gender stereotyping (Miller, Lurye, Zosuls, & Ruble, 2009), and begin to develop their own stereotypes related to gender-typed activities, careers, and roles (Blakemore, 2003). While these explicit stereotypes decline in middle childhood, implicit measures have revealed that gender stereotypes persist beyond this point into late childhood and adolescence (Steffens & Jelenec, 2011; Wilbourn & Kee, 2010). One key application of the extensive research conducted on developing gender stereotypes has been to understand the development of gender stereotypes related to STEM.

Gender stereotypes related to STEM ability have important consequences for STEM engagement and motivation in later life. Gendered stereotypes regarding who can succeed in STEM threaten the career choices of women and can help explain why women who do pursue a STEM career may eventually leave their chosen field (Beasley & Fischer, 2012; Cundiff, Vescio, Loken, & Lo, 2013). Evidence suggests that science ability can be viewed as gender innate (Mascret & Cury, 2015); that is, that men are simply “born” to succeed in the STEM world. Such stereotypes have damaging consequences related to women's STEM self-efficacy and career motivation (Cundiff et al., 2013; Schuster & Martiny, 2017). For example, research with adolescents has demonstrated that STEM stereotypes are a significant predictor of STEM self-efficacy, which in turn predicts future career aspirations (Garriott, Hultgren, & Frazier, 2017).

These stereotypes emerge in childhood, with recent evidence demonstrating that children between three- and five-years-old show less support for counter-stereotypical STEM career choices (e.g., a girl who wanted to be an engineer; Mulvey & Irvin, 2018). Similarly, using both implicit and explicit stereotype measures, six- to ten-year-old children have been shown to hold the stereotype that math is for boys, with male participants identifying more strongly with math on both types of measure (Cvencek et al., 2011). This is especially concerning in light of meta-analytic evidence that suggests that girls and boys do not, in fact, perform differently in measures of math ability (Lindberg, Hyde, Petersen, & Linn, 2010). These stereotypes then are not founded in any real gender performance or ability differences (Wang, Eccles, & Kenny, 2013), and yet can lead to the social exclusion of girls and women from childhood through to adulthood.

While children's gender stereotypes emerge early, in middle childhood there is evidence that ideas related to gender roles become more flexible (Liben & Bigler, 2002). That is, children begin to understand that men and women can fulfill different roles, independent of their gender. Despite this, in middle childhood, there remains a belief that ‘masculine’ careers have a higher status than ‘feminine’ careers (Liben & Bigler, 2002). Therefore, it is important to examine these stereotypes from early childhood into middle childhood. In early adolescence, gender attitude and behavior flexibility continue to develop, with an intensification of the need to conform to gender roles especially apparent amongst males (Bartini, 2006). Given this developing gender flexibility between early childhood and early adolescence, the present work, for the first time, examines gender stereotypes in the context of STEM across this developmental span. As evidence has shown that gender role intensification is particularly apparent amongst males, we expected to observe greater adherence to STEM stereotypes amongst male, compared to female participants.

### Informal settings & educators

Ideas about who can succeed in STEM have a powerful influence on the representation of marginalized groups in the science workforce, with such stereotypes leading to women not choosing STEM careers, or leaving the field early (Beasley & Fischer, 2012; Cundiff et al., 2013). However, greater representation can in turn affect ideas of who can succeed in STEM. Data from over 60 countries has demonstrated that where there is a higher representation of women employed in tertiary STEM education positions (community college and above), explicit and implicit national gender stereotypes are more flexible (Miller, Eagly, & Linn, 2015).

Stereotypes about STEM may be fostered or challenged in formal learning environments, as youth have frequent opportunities to learn STEM in formal settings. Although STEM ecosystems change from primary school to high school, the Next Generation Science Standards highlight the importance for prioritization of the same core components (crosscutting concepts, science and engineering practices and disciplinary core ideas) across developmental periods (NGSS Lead States, 2013; WISE, 2019). However, this does not mean that the same messages about STEM stereotypes are communicated in different settings or in different developmental periods.

Evidence from formal learning settings indicates that interactions with counter-stereotypical STEM educators can challenge the consequences of stereotypes. For example, in computer science classrooms, girls report greater concern about being negatively stereotyped than boys when they have a male teacher; however, the presence of a female teacher alleviates this concern (Master, Cheryan, & Meltzoff, 2014). Male students can also be positively influenced by interactions with counter-stereotypical teachers and peers. In one examination of an engineering classroom, male participants (17–18-years-old) who were taught by female teachers, in classrooms with a higher number of female peers, demonstrated a reduction in male-biased gender stereotypes over a year compared to those who had male teachers and less female peer representation (Riegle-Crumb et al., 2017). The number of female teachers in STEM in the US has increased from 43% in 1988 to 64% in 2012 (Nguyen & Redding, 2018), suggesting that, in formal settings, children increasingly have access to opportunities to interact with STEM educators who challenge gendered norms. However, a visual analysis of STEM materials used in formal classrooms confirmed that science learning materials reinforce stereotypes associating science with men (Kerkhoven, Russo, Land-Zandstra, Saxena, & Rodenburg, 2016). Thus, even if children are provided with counter stereotypic STEM teachers, they may still receive implicit input suggesting that science is for boys and men. Importantly, children's and adolescents' STEM learning does not solely take place in formal settings, and less is known about how interactions in informal settings may influence ideas about gender equity in STEM.

Informal STEM learning settings (ISLS) provide opportunities for children and adolescents to learn outside of the classroom (Dierking & Falk, 2018). Research indicates that a great deal of learning about STEM and who STEM is “for” occurs in in informal STEM settings (National Research Council, 2009). Informal STEM learning has been shown to promote positive science attitudes (National Research Council, 2009), which may be especially useful in countering the pervasive stereotypes about ability and STEM. This may be in part because these sites also offer opportunities to interact with a variety of STEM educators, who may represent a broader cross-section of society than the small number of STEM teachers with whom children and adolescents interact in formal settings. While research on learning in informal settings has documented that these informal environments can scaffold science learning for youth from non-dominant backgrounds, such as girls and women (National Research Council, 2009), less is known about if and how quickly informal STEM learning opportunities can promote attitudes countering STEM gender stereotypes. Informal settings provide an opportunity to explore the dosage that is required to observe the effects of diverse representation on gender stereotyping. Typically, an interaction with an educator in an informal site will be much shorter and shallower than the relationship formed between student and teacher in formal educational settings. We know that long-form interactions *can* challenge the consequences of STEM gender stereotyping. However, we do not yet know what the minimal form of interaction that can serve to challenge STEM gender stereotypes looks like. Therefore, a second aim of this study was to examine whether a short interaction with a counter-stereotypical educator had any bearing on responses to STEM gender stereotypes.

### The present study

The present study extends existing work related to STEM gender stereotypes by examining the development of these stereotypes between early childhood and adolescence. We examined children from early childhood where explicit gender stereotypes about STEM domains have been shown to emerge (Cvencek et al., 2011), through middle childhood where gender role flexibility increases and rigid gender stereotypes begin to decline (Liben & Bigler, 2002). Further, we extend this examination into adolescence, where we know that these gender stereotypes have a measurable impact upon STEM engagement and motivations (Song, Zuo, Wen, & Yan, 2017). In particular, we focus on whether explicit stereotypes change in to adolescence and in what way. Further, this study provides the first examination of the potential influence of educators in ISLS on children's and adolescents' explicit STEM gender stereotypes. Participants were visitors to one of four ISLS, who were surveyed after visiting a pre-selected gallery or exhibit, where they either interacted with an educator, or no educator was present. We measured participants' gender stereotype awareness, endorsement, and flexibility.

### Hypotheses

H1 Responses to STEM gender stereotype awareness, endorsement and flexibility measures were expected to become more equitable (i.e., less biased towards one gender group over another) between early childhood, middle childhood, and adolescence.

H2 Female participants' responses to STEM gender stereotype awareness, endorsement and flexibility measures were expected to be more equitable (i.e., less biased towards one gender group over another) than male participants' responses.

For both of the above hypotheses, when participants' responses were less equitable we expected this to be due to an increase in male-biased responses.

We also examined whether participants' STEM gender stereotype awareness, endorsement and flexibility varied following an interaction with a female educator, compared to a male educator, or a situation where no educator interaction took place. Given the strength of STEM gender stereotypes, it was an open question as to whether a short-form interaction with an educator would have an effect on stereotypes or not.

## Method

### Participants

Participants (*n* = 997, female = 572) were recruited from four informal science learning sites. These included a zoo (*n* = 239), an aquarium (*n* = 320), and a children's science museum (*n* = 147) located in the Southeastern USA, as well as a family science museum (*n* = 291) in the Midlands of the United Kingdom. Participants were divided into three age groups: early childhood (*n* = 407, M^age^ = 6.61, SD = 1.17, min. = 5-years, max. = 8-years), middle childhood (*n* = 343, M^age^ = 9.96, SD = 0.84, min. = 7-years, max. = 11-years), and adolescence (*n* = 220, M^age^ = 13.86, SD = 1.88, min. = 12-years, max. = 18-years). 66% of participants identified as members of the ethnic majority group of the country of testing (White British in the UK sites, White European-American in the US sites). See supplemental materials for a full breakdown of the ethnicity of the sample. Parental consent and child assent were obtained for all participants.

## Procedure

All measures were approved by the [INSTITUTION BLINDED FOR REVIEW] IRB as part of the project [BLINDED FOR REVIEW]. The protocol was completed in the ISLS using either online survey software (Qualtrics, Provo, UT) on a tablet computer, or in hard copy. In both cases the same measures were utilized. Younger children who were less confident in their reading ability completed the survey in a one-to-one interview format with an experimenter. Older children who were more confident in their reading ability completed the survey individually. In order to combat potential self-presentational concern in the survey we stressed to participants in a verbal brief that their answers were totally anonymized and that their personal details were not linked to their answers.

Participants were recruited on site by experimenters, and offered either an electronic gift card, gift shop voucher or gift bag (depending on funding agency or institutional policy) worth $/£5 in exchange for completing a questionnaire. Participants were part of family groups visiting the site, consisting of at least one adult and one child. All participants were approached at the exit of pre-selected galleries or exhibits. These exhibits were chosen in conjunction with ISLS staff, recognized as popular areas of the site where educators were regularly stationed. During the participants' time at the exhibit, there was either an educator present (*n* = 417), or no educator present (*n* = 540). The presence of an educator varied based on the ISLS' own scheduling of educators, so visitors were assigned to either the educator or no educator condition based upon this schedule. Participants who visited the gallery in the presence of an educator were in turn assigned to the male educator (*n* = 165) and female educator (*n* = 205) conditions based on the ISLS scheduling of educators. Of these participants, 82% reported that their interaction lasted 5 min or less.

### Materials

The measures presented here were part of a larger questionnaire examining the influence of youth educators in ISLS that also included measures related to STEM learning, interest, and STEM ethnic stereotypes.

### Gender stereotype measure

The gender stereotype measure utilized here was adapted from Liben and Bigler (2002). Participants were asked to select whether they thought boys, girls or both boys and girls best fit a number of questions.

*Stereotype Awareness:* “Who do you think is usually good at Science?”, “Who do you think is usually good at Technology?”, “Who do you think is usually good at Engineering?”, “Who do you think is usually good at Math?” (‘Boys’, ‘Girls’, ‘Both Boys and Girls’).

*Stereotype Endorsement:* “Who do you think should be good at Science?”, “Who do you think should be good at Technology?”, “Who do you think should be good at Engineering?”, “Who do you think should be good at Math?” (‘Boys’, ‘Girls’, ‘Both Boys and Girls’).

*Stereotype Flexibility:* “Who do you think can be good at Science?”, “Who do you think can be good at Technology?”, “Who do you think can be good at Engineering?”, “Who do you think can be good at Math?” (‘Boys’, ‘Girls’, ‘Both Boys and Girls’).

### Data analytic plan

Principal components analysis was conducted in order to determine whether the 12 items that comprised the gender stereotype measure would load on to three distinct factors representing awareness, endorsement and flexibility. For these 12 items, the Kaiser-Meyer-Olkin measure of sampling adequacy was 0.86, and Bartlett's test of sphericity was significant, *χ*^*2*^ (66) = 4525.46, *p* < .001). Further, each item had communalities above 0.3, suggesting that each item shared variance with other items. These indicators suggested the 12 items were suitable for PCA.

PCA with varimax rotation was conducted, revealing three factors with eigenvalues above 1, cumulatively explaining 58.21% of the variance. Each item in this analysis had primary loadings on its factor of over 0.5. These three factors fit with the proposed stereotype awareness, endorsement and flexibility labels (Liben & Bigler, 2002).

Given the results of this PCA, responses to the gender stereotype questions were summed to create three 0–4 stereotype response scales for awareness, endorsement and flexibility, as follows:

*Equitable Response* scale (0 = no ‘both boys and girls’ responses given, 4 = ‘both boys and girls’ response given for all questions within the measure).

*Male Bias* scale (0 = no ‘boys’ responses given, 4 = ‘boys’ response given for all questions within the measure). *Female Bias* scale (0 = no ‘girls’ responses given, 4 = ‘girls’ response given for all questions within the measure).

So, for the three sub-scales (stereotype awareness, endorsement and flexibility) an equitable response score, male-bias score, and female-bias score were calculated for each participant. These scores were treated as a within-subjects repeated measures “Stereotype Response” factor in our analyses. This approach allowed us to assess whether participants were showing male or female bias when they were not providing equitable responses.

To account for the multi-site nature of our data we calculated intra-class correlation coefficients (ICC) across sites and exhibits within sites. For both site (ICC = 0.003) and exhibit within site (ICC = 0.004) the low ICC did not suggest that multi-level modeling was the most appropriate analytic approach.

In order to determine whether the presence of an educator was influential, we conducted a series of 3 (Participant Age; Early Childhood, Middle Childhood, Adolescence) × 2 (Participant Gender; Female, Male) × 2 (Educator Presence; Educator, No Educator) × 3 (Stereotype Response; Equitable, Male Bias, Female Bias) ANOVAs with repeated measures on the last factor with stereotype awareness, endorsement and flexibility as dependent variables. In order to examine whether educator gender was influential *within* the sample who interacted with an educator we also conducted a series of 3 (Participant Age; Early Childhood, Middle Childhood, Adolescence) × 2 (Participant Gender; Female, Male) × 2 (Educator; Female, Male) × 3 (Stereotype Response; Equitable, Male Bias, Female Bias) ANOVAs with repeated measures on the last factor with stereotype awareness, endorsement and flexibility as dependent variables. Where appropriate, simple main effects comparisons were conducted with Bonferroni corrections for multiple comparisons applied.

## Results

Across the stereotype awareness, endorsement and flexibility measures, analyses did not reveal any effect of educator, nor of educator gender, so these variables were dropped from the analyses described below. All the below analyses report the results of 3 (Participant Age; Early Childhood, Middle Childhood, Adolescence) × 2 (Participant Gender; Female, Male) × 3 (Stereotype Response; Equitable, Male Bias, Female Bias) ANOVAs with repeated measures on the last factor.

### STEM stereotype awareness

Analyses revealed a significant interaction between stereotype response and participant age group, *F*(4, 1828) = 14.90, *p* < .001, *η*^*2*^ = 0.03. Participants in early childhood gave less equitable responses (*M* = 1.96, SD = 1.36) than those in middle childhood (*M* = 2.36, SD = 1.37, *p* = .001) and adolescence (*M* = 2.70, SD = 1.41, *p* < .001). Participants in middle childhood gave less equitable responses than those in adolescence (*p* = .008). Between early childhood and adolescence participants were more likely to say that ‘both boys and girls’ were usually good at STEM.

In early childhood there was greater male bias (*M* = 1.33, SD = 1.11) than in adolescence (*M* = 0.95, SD = 1.15, *p* < .001). There was no difference in male bias between early childhood and middle childhood (*M* = 1.12, SD = 1.11, *p* = .10). Similarly, there was no difference in male bias between middle childhood and adolescence (*p* = .11). Finally, in early childhood there was greater female bias (*M* = 0.70, SD = 0.99) than in middle childhood (*M* = 0.52, SD = 0.77, *p* = .004) and adolescence (*M* = 0.34, SD = 0.62, *p* < .001). There was no difference in female bias between middle childhood and adolescence (*p* = .06). In early childhood, compared to middle childhood or adolescence, participants were more likely to say that ‘boys’ or ‘girls’ were usually good at STEM.

**Fig. 1:**
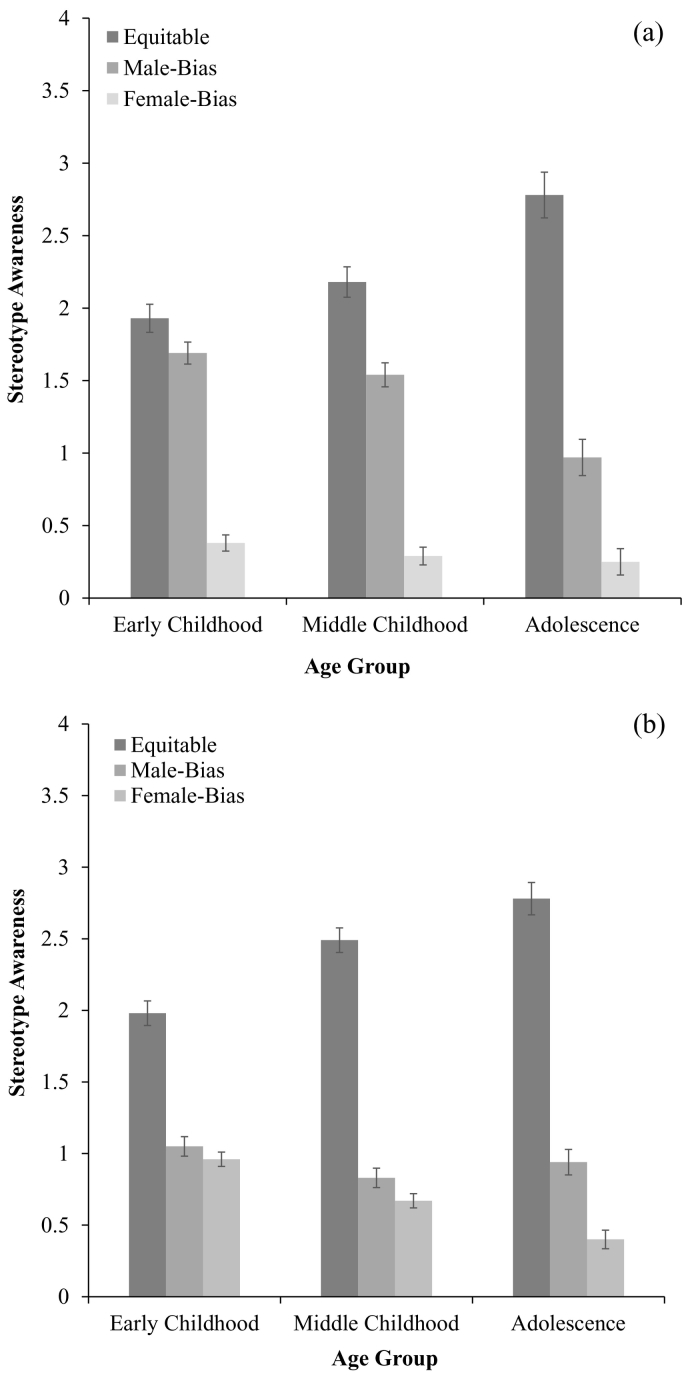
a. Male participants' stereotype awareness as a function of participant age group (w. standard error bars). b. Female participants' stereotype awareness as a function of participant age group (w. standard error bars).

This effect was qualified by an interaction between stereotype response, age group and gender, *F*(4, 1828) = 3.24, *p* = .01, *η*^*2*^ = 0.007 (see Fig. 1a and b). Crucially, in middle childhood, female participants (*M* = 2.49, SD = 1.34) gave more equitable responses than male participants (*M* = 2.16, SD = 1.39, *p* = .02). In this age group, male participants (*M* = 1.55, SD = 1.24) demonstrated greater male bias than female participants (*M* = 0.83, SD = 0.90, *p* < .001). Similarly, female participants (*M* = 0.67, SD = 0.89) demonstrated greater female bias than male participants (*M* = 0.29, SD = 0.47, *p* < .001). However, for male participants in this age range, there was greater male bias than female bias (*p* < .001), while female participants did not demonstrate greater female bias than male bias (*p* = .18).

In middle childhood, when male participants did not say that both boys and girls were usually good at STEM, they were more likely to respond that ‘boys’ are usually good at STEM than they were to say that girls are usually good at STEM. Female participants, by comparison, were not more likely to say that ‘girls’ were usually good at STEM than they were to say that boys are usually good at STEM.

### STEM stereotype endorsement

**Fig. 2:**
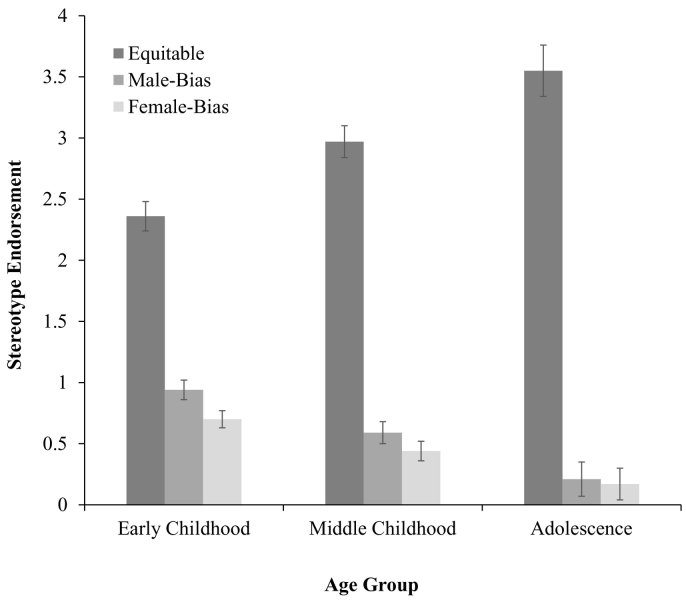
Stereotype endorsement as a function of participant age group (w. standard error bars).

Analyses revealed a significant interaction between stereotype response and participant age group, *F*(4, 1820) = 36.91, *p* < .001, *η*^*2*^ = 0.08 (see Fig. 2). Participants in early childhood gave less equitable responses (*M* = 2.36, SD = 1.56) than those in middle childhood (*M* = 2.96, SD = 1.43, *p* < .001) and adolescence (*M* = 3.55, SD = 1.07, *p* < .001). In turn, participants in middle childhood demonstrated less equitable responses than those in adolescence (*p* < .001). Again, with age, participants were more likely to respond that ‘both boys and girls’ *should* be good at STEM.

In early childhood there was greater male bias (*M* = 0.94, SD = 1.11) than in middle childhood (*M* = 0.59, SD = 1.00, *p* < .001) and adolescence (*M* = 0.29, SD = 0.75, *p* < .001). There was a further difference in male bias between middle childhood and adolescence (*p* = .001). Finally, in early childhood there was greater female bias (*M* = 0.70, SD = 1.03) than in middle childhood (*M* = 0.44, SD = 0.91, *p* < .001) and adolescence (*M* = 0.16, SD = 0.52, *p* < .001). Similarly, there was a difference in female bias between middle childhood and adolescence (*p* = .001). In early childhood participants exhibited greater male or female bias in endorsement (i.e., who ‘should’ be good at STEM) and less equitable responses than those in middle childhood or adolescence.

**Fig. 3:**
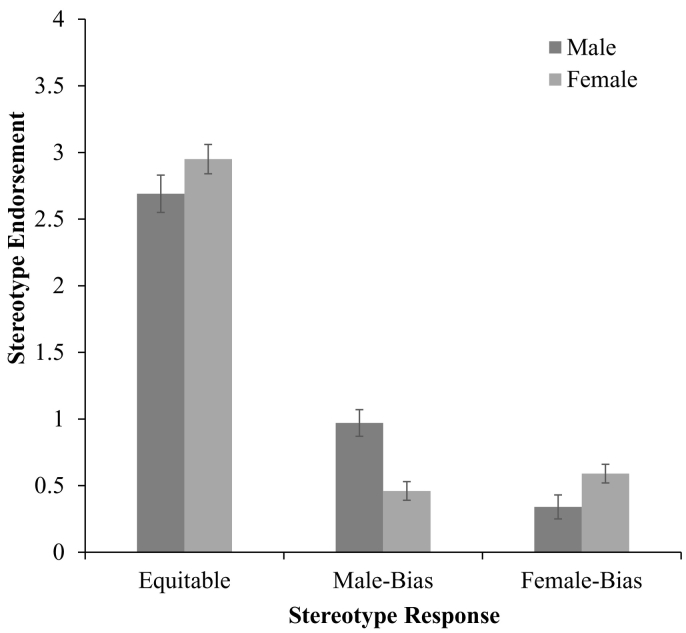
Stereotype endorsement as a function of participant gender (w. standard error bars).

Further, we observed a significant interaction between stereotype response, and participant gender, *F*(2, 1820) = 15.76, *p* < .001, *η*^*2*^ = 0.02 (see Fig. 3). Female participants (*M* = 2.94, SD = 1.44) gave more equitable responses than male participants (*M* = 2.67, SD = 1.55, *p* = .02). Further, male participants demonstrated greater male bias (M = 0.98, SD = 1.26) than female participants (M = 0.47, SD = 0.78, *p* < .001). Finally, female participants (*M* = 0.59, SD = 1.04) gave greater female bias responses than male participants (*M* = 0.35, SD = 0.69, *p* = .001). However, while male participants demonstrated greater male bias than female bias (*p* < .001), there was no difference between male and female bias for female participants (*p* = .23). Similarly to stereotype awareness, although here independent of age, male participants endorsed an in-group biased perspective of who ‘should’ be good at STEM by reporting that boys should be good at STEM more than they reported that girls should be good at STEM. In contrast female participants did not report that girls should be good at STEM more than they reported that boys should be good at STEM.

### STEM stereotype flexibility

**Fig. 4:**
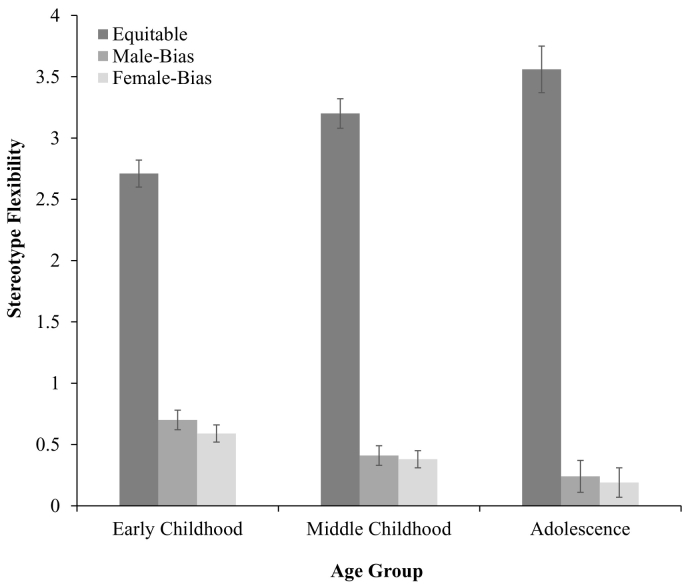
Stereotype flexibility as a function of participant age group (w. standard error bars).

Analyses revealed a significant interaction between stereotype response and participant age group, *F*(4, 1826) = 23.70, *p* < .001, *η*^*2*^ = 0.05 (see Fig. 4). Participants in early childhood gave less equitable responses (*M* = 2.71, SD = 1.48) than those in middle childhood (*M* = 3.21, SD = 1.33, *p* < .001) and adolescents (*M* = 3.54, SD = 1.09, *p* < .001). There was a further difference in equitable responses between middle childhood and adolescence (*p* = .003). Again, with age participants were more likely to respond that ‘both boys and girls’ *can* be good at STEM.

In early childhood there was greater male bias (*M* = 0.69, SD = 1.01) than in middle childhood (*M* = 0.42, SD = 0.89, *p* < .001) and adolescence (*M* = 0.25, SD = 0.75, *p* < .001). Further, there was greater male bias in middle childhood than in adolescence (*p* = .03). In early childhood there was greater female bias (*M* = 0.59, SD = 1.03) than in middle childhood (*M* = 0.37, SD = 0.84, *p* = .002) and adolescence (*M* = 0.20, SD = 0.67, *p* = .002). In middle childhood there was greater female bias demonstrated than in adolescence (*p* = .05). In early childhood, compared to middle childhood or adolescence, participants were more likely to say that ‘boys’ or ‘girls’ can be good at STEM.

**Fig. 5:**
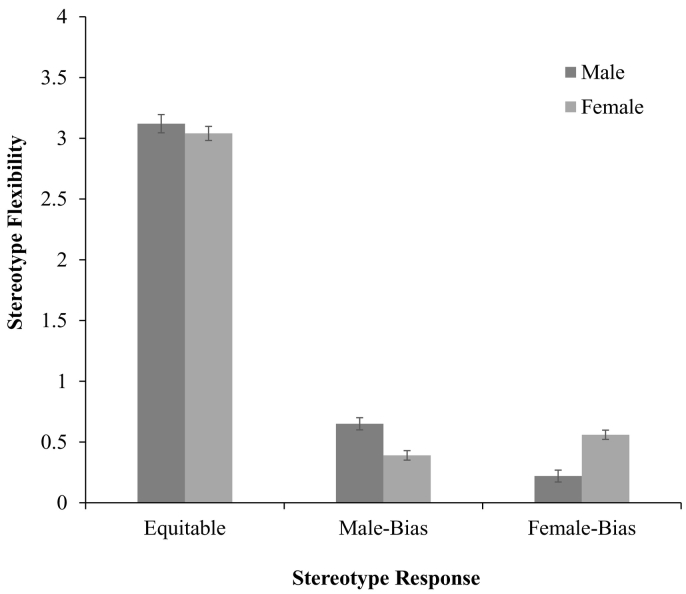
Stereotype flexibility as a function of participant gender (w. standard error bars).

Finally, we observed a significant interaction between stereotype response and participant gender, *F*(2, 1826) = 9.75, *p* < .001, *η*^*2*^ = 0.01 (see Fig. 5). There was no difference in equitable responses for male (*M* = 3.12, SD = 1.38) and female participants (*M* = 3.04, SD = 1.40, *p* = .23). However, male participants (*M* = 0.65, SD = 1.13) gave greater male-biased responses than female participants (*M* = 0.39, SD = 0.75, *p* = .001). Similarly, female participants (*M* = 0.59, SD = 1.03) gave greater female-biased responses than male participants (*M* = 0.22, SD = 0.63, *p* < .001). For flexibility responses, both male participants (*p* < .001) and female participants (*p* = .007) demonstrated in-group in who they thought ‘can’ be good at STEM. When they didn't say that ‘both boys and girls’ can be good at STEM, participants from each gender group demonstrated in-group bias by saying that their own gender can be good at STEM.

## Discussion

This study demonstrated a developmental trend in increasingly equitable responses (i.e., ‘both boys and girls’ rather than just ‘boys’ or ‘girls’) to STEM gender stereotype measures between early childhood, middle childhood and especially in to adolescence. Where equitable responses were not given in early childhood, participants were more likely to display male and female in-group bias, suggesting that in this age range there is a belief that one's gender in-group usually out-performs other groups. Male bias was observed when male participants were asked who they thought ‘should’ be good at STEM (i.e., stereotype endorsement), but a similar in-group bias effect was not apparent amongst female participants when asked the same question. Participants' responses to these stereotype measures were not influenced by an interaction with an educator, relative to a no educator condition, nor were they influenced by the gender of the educator. Crucially these findings demonstrate a pattern of increasingly equitable responses to explicit STEM gender stereotype measures between early childhood and adolescence.

STEM is one area in which gender stereotypes about ability sustain the status of men (Jost & Kay, 2005; Koenig, 2018). Evidence has shown that children are impacted by such stereotypes in school contexts (Master et al., 2014). Consistent with this work the present findings demonstrate these stereotypes are sustained outside of the classroom. In early childhood, when they did not provide an equitable response, participants showed a greater tendency to say that members of their gender in-group (either ‘boys’ or ‘girls’) were *usually* good at STEM, *can be* good at STEM and *should be* good at STEM. This finding extends our knowledge of STEM gender stereotypes in childhood by demonstrating that high prevalence of in-group bias during childhood (Raabe & Beelmann, 2011) extends in to the STEM domains. Rather than internalizing broader societal stereotypes about male dominance in STEM, younger female participants are more likely to report that members of their own group *should* and *can be* good at STEM. This is a promising finding that highlights early childhood as a key window in which educational interventions aimed at fostering female engagement with STEM may have greater impact.

By comparison, in middle childhood and adolescence, equitable responses to stereotype measures become the most prominent response type. Where in-group bias is apparent, it is seen amongst male participants in middle childhood when asked who is *usually* good at STEM. These participants were more likely to express male-bias than female-bias. While there is a trend towards more equitable responses, the in-group bias of early childhood is still apparent by middle childhood. This is not the case for female participants in middle childhood, who did not show the same in-group bias that girls in early childhood did. This finding likely reflects the influence of entering the formal schooling context, where boys' ideas about their STEM ability are reinforced and girls may be dissuaded from their belief that they *can* and *should be* good at STEM. This finding further stresses the importance of implementing interventions in schools as early as possible to challenge male-bias where it does exist.

In adolescence, female participants are just as likely as male participants to say that ‘both boys and girls’ are *usually*, *can*, and *should be* good at STEM. In spite of their self-reported equitable STEM ability stereotypes, adolescent girls lose interest in STEM and are less well represented by the time they reach college (Shapiro & Williams, 2012; WISE, 2018). This suggests that less equitable explicit stereotypes held by adolescent girls themselves are not necessarily the root cause of this loss of interest. Instead, research should look to more implicit stereotypes, or the influence of stereotypes held by STEM gatekeepers that may invoke stereotype threat and in turn reduce interest, as causes of this drop in interest. Overall, it is promising that, separate from the well-documented consequences of STEM gender stereotypes, children and adolescents are increasingly likely to favor an explicitly equitable view of STEM ability.

Here we have focused on relative differences in male and female-biased responses to gender stereotype measures. However, the overwhelming response from early childhood through to adolescence was that ‘both boys and girls’ *usually*, *should*, and *can be* good at STEM. This age trend reflects the increase between middle childhood and adolescence in equitable thinking about gender stereotypes that has been documented in areas outside of STEM (Alfieri, Ruble, & Higgins, 1996; Banse, Gawronski, Rebetez, Gutt, & Bruce Morton, 2010). Of course, this finding does not indicate that STEM gender stereotypes about male ability do not exist, but rather that from a young age, children are more likely to endorse the explicit position that both boys and girls are equally talented in STEM.

Participant gender also played a role in stereotype responses independent of participant age. When asked who was ‘usually’ good at STEM (stereotype awareness), male participants were more likely to show male bias than were female participants, and vice versa for female bias. Further, male dominance beliefs were reflected in the STEM endorsement (‘should’) and flexibility (‘can’) measures, where male participants demonstrated greater male bias than did female participants. Traditional stereotypes emphasize the success and ability of men in STEM (Carli, Alawa, Lee, Zhao, & Kim, 2015). However, in the present work, children's and adolescents' stereotype responses did not always align with this idea of male superiority. This is an interesting finding, and an important follow up question is to determine why female participants from late childhood in to adolescence move towards more equitable responses while male participants continue to demonstrate some form of male-biased responding.

This is especially surprising as while boys may observe greater instances of men employed in STEM careers, male-biased responses are not reflective of what they observe in terms of gender and STEM performance in schools. In fact, in their STEM classes, they are more likely to see no discernible STEM ability difference between boys and girls (Hyde, Fennema, & Lamon, 1990; Hyde, Lindberg, Linn, Ellis, & Williams, 2008; Lindberg et al., 2010). A recent meta-analysis of over 1.6 million high school students' STEM grades awarded between 1931 and 2013 revealed no difference between boys' and girls' performance (O'Dea, Lagisz, Jennions, & Nakagawa, 2018). This may help explain relative in-group bias in gender stereotype awareness but does not explain why female participants move away from this in-group bias more than male participants.

Together these findings stress the importance of interventions throughout the developmental span observed in the present study. Developing methods to foster early beliefs about women's STEM ability during early childhood, while not perpetuating ideas of adult male dominance, is an essential first step for those interested in equal representation in STEM. For example, a focus in early childhood on the many successful female scientists, mathematicians and innovators from the fields of engineering and technology could be a key strategy to strengthen the idea that women usually, can and should do well in these domains. At the same time, by middle childhood and adolescence it is important to target interventions towards boys, who (when not giving equitable responses) in this age range are more likely to show in-group bias than girls. By adolescence, where critical consciousness and an understanding of inequality emerges (Diemer & Rapa, 2016), education regarding systematic under-representation in STEM may be an effective tactic in challenging male-bias where it does exist.

In the present study we did not observe an influence of educator interaction on responses to gender stereotype measures. This offers an important caveat to previous work examining the role of female teachers and role models (Master et al., 2014). Based on these findings, it is likely that both interaction quantity and quality play a role in determining whether a STEM expert will influence stereotypes. Short form interactions such as those in an ISLS do not appear to be effective in the same way year-long interactions with a class teacher are.

In the present work, the majority of participants reported that their interaction with an educator lasted 5 min or less. It is an open question as to whether there is a middle ground in interaction quantity; for example, it is possible that a day or week-long STEM summer school with high female representation would have beneficial effects for both male and female children. Future research should aim to probe at what dosage we begin to see effects of diverse representation on gender stereotypes. Further, we know that role models who challenge STEM stereotypes (e.g., a computer scientist who enjoys playing sports rather than watching *Star Wars*; Cheryan, Siy, Vichayapai, Drury, & Kim, 2011), can change women's beliefs about their potential STEM success, regardless of the gender of the role model. Understanding the role of stereotype-confirmatory behaviors that educators in ISLS may embody, independent of the educator's gender, may help us to understand when and how these interactions can serve to challenge gender stereotypes.

## Limitations & future directions

One possible explanation for the lack of educator effects is that, overall, the mean responses to the stereotype measures were equitable, and where differences in male-bias were observed, the effect sizes were relatively small. We know that by middle childhood, individuals understand the possible ramifications of displaying explicit out-group prejudice and temper their explicit displays of bias accordingly (Fitzroy & Rutland, 2010; Rutland, Cameron, Milne, & McGeorge, 2005). It is possible that in the present work, the more equitable responses seen to emerge between middle childhood and early adolescence are reflective of this self-presentational concern.

In order to account for this possible explanation, future research should utilize more indirect or implicit measures of gender stereotyping in the context of STEM to understand whether differences in middle childhood and adolescence are driven by self-presentational concerns, and whether interacting with an educator can influence STEM stereotypes. For example, participants could be asked who they would include in a STEM after-school club, or how they would allocate STEM-relevant resources between gendered groups. Such measures will help to identify whether beliefs about STEM and gender during middle childhood and adolescence are influenced by experiences with different educators in informal science learning settings.

Another way to examine the broader influence of these stereotypes would be to explore whether these gender stereotypes are related to self-reported STEM ability. While there was a trend towards equitable responses to our stereotype measures, it is not clear whether this same equitable pattern would be reflected in questions such as “how good are *you* at STEM?”. Such questions would provide an interesting insight in to how stereotypes about STEM are internalized, and perhaps even predict self-reported perceptions of individual ability.

One interesting element of the present sample is that they likely represent a group whose parents or guardians have higher science capital and have self-selected to visit an ISLS with their children (Archer, Dawson, DeWitt, Seakins, & Wong, 2015). This group may represent a sub-sample of the population who wish to challenge STEM gender stereotypes and impart such beliefs to their children. There is some evidence from other kinds of museums that visitors are more politically liberal and open to other cultures and the lifestyles of others (DiMaggio, 1996). However, less is currently known about similar characteristics in science center visitors, compared to non-visitors. An essential step for future research is to explore such beliefs amongst science center visitors compared to a non-visitor sample. A measure of science capital, as well as other indicators of the STEM context in which children are living (e.g. access to STEM-focused toys, books, and courses) will provide further insight in to the development of STEM gender stereotypes. Further, taking in to consideration the clustering of family groups as a level of analysis could provide insight in to how parent and sibling dynamics impact upon children's STEM gender stereotypes.

## Conclusion

This study extends previous work that has examined STEM gender stereotypes at various developmental stages by showing a clear trend towards more equitable ideas about STEM and gender from early childhood, through middle childhood, into adolescence. From an early childhood in-group bias, participants move to more equitable awareness of stereotypes, along with endorsement and flexibility. However, these results also reveal that when not giving equitable responses, male participants can endorse a male-biased perspective of STEM. Gender stereotypes negatively influence perceived self-efficacy, course enrolment and career length for women in STEM. Developing ways to challenge conceptions about who should be able to succeed in science is a key focus for educators and policy-makers, and informal science learning settings are likely to be an important context where strategies can be developed to promote more equitable beliefs around STEM and gender.

## Funding

This work was supported in the US by the National Science Foundation [grant number: DRL-1831593]; and collaboratively in the UK by the Wellcome Trust [grant number: 206259/Z/17/Z] and the Economic and Social Research Council.

The authors would like to thank our practitioner partners at Riverbanks Zoo & Gardens (USA), Thinktank Science Museum (UK), Virginia Aquarium (USA), and EdVenture (USA). In particular we would like to thank Zain Iqbal for their help during data collection. We would also like to thank all the visitors to these sites who participated.

## Appendix A Supplementary data

Supplementary material Image 1
