# Supplementary material for: STEM gender stereotypes from early childhood through adolescence at informal science centers
Source: J Appl Dev Psychol. 2020 Mar-Apr;67:101109. doi: 10.1016/j.appdev.2020.101109 (PMC7104893; doi:10.1016/j.appdev.2020.101109)
Supplement: Supplementary file 1 — Supplementary material [file mmc1.docx]

Supplemental Table 1: Sample Ethnicity

| Ethnicity | Frequency | Percentage |
| --- | --- | --- |
| African-American | 94 | 9.4 |
| American Indian/Native American | 15 | 1.5 |
| Asian/Asian-American | 35 | 3.5 |
| Bi-Racial/Multi-Racial (US) | 52 | 5.2 |
| Black British | 6 | 0.6 |
| Chinese British | 7 | 0.7 |
| Hispanic/Latinx | 27 | 2.7 |
| Indian British | 13 | 1.3 |
| Missing | 32 | 3.2 |
| Mixed Race/Dual-Heritage (UK) | 19 | 1.9 |
| Native Hawaiian/Pacific Islander | 11 | 1.1 |
| Other (UK) | 13 | 1.3 |
| Other (US) | 36 | 3.6 |
| Pakistani British | 19 | 1.9 |
| White British | 207 | 20.8 |
| White/European American | 411 | 41.2 |
